# Environmental phthalate and chronic kidney disease: Epidemiology, mechanistic pathways, and clinical implications

**DOI:** 10.1016/j.isci.2026.118139

**Published:** 2026-11-05

**Authors:** Chengsheng Wang, Hongliang Cao, Qianhui Li, Yang Tan, Zheqi Li, Shuangfeng Chen, Song Wang

**Affiliations:** 1 Department of Urology, The First Hospital of Jilin University, Changchun 130021, China; 2 Key Laboratory of Pathobiology, Ministry of Education, Jilin University, Changchun, China; 3 Department of Urology, The Sixth Affiliated Hospital of Xinjiang Medical University, Urumqi, China

**Keywords:** phthalates, chronic kidney disease, CKD, urinary biomarkers, oxidative stress and inflammation

## Abstract

Chronic kidney disease (CKD) remains a major global health problem, and early detection and risk control are still limited. Phthalates—widely used plasticizers with endocrine-disrupting effects—have been increasingly implicated in CKD-related outcomes. Human studies have reported associations between urinary phthalate metabolites and kidney-related markers, commonly reflected by the albumin-to-creatinine ratio (ACR) and estimated glomerular filtration rate (eGFR), although the direction and magnitude of these associations vary by metabolite, population, study design, and outcome definition. Mechanistic evidence is currently dominated by studies of di-(2-ethylhexyl) phthalate (DEHP) and its metabolite mono-(2-ethylhexyl) phthalate (MEHP), suggesting potential involvement of oxidative stress, inflammation, regulated cell death, epithelial-mesenchymal transition (EMT), and extracellular-matrix (ECM) remodeling in experimental models. However, the extent to which these mechanisms apply to other phthalates or alternative plasticizers remains uncertain. The evidence remains heterogeneous across populations and exposure contexts, and key limitations include its observational nature, exposure-measurement bias, reverse causality, and uncertainty regarding temporality and mixture effects. Future longitudinal, pharmacokinetic, and intervention studies are needed to determine whether phthalate biomarkers have clinically meaningful prognostic or predictive utility.

## Graphical abstract

**Figure:**
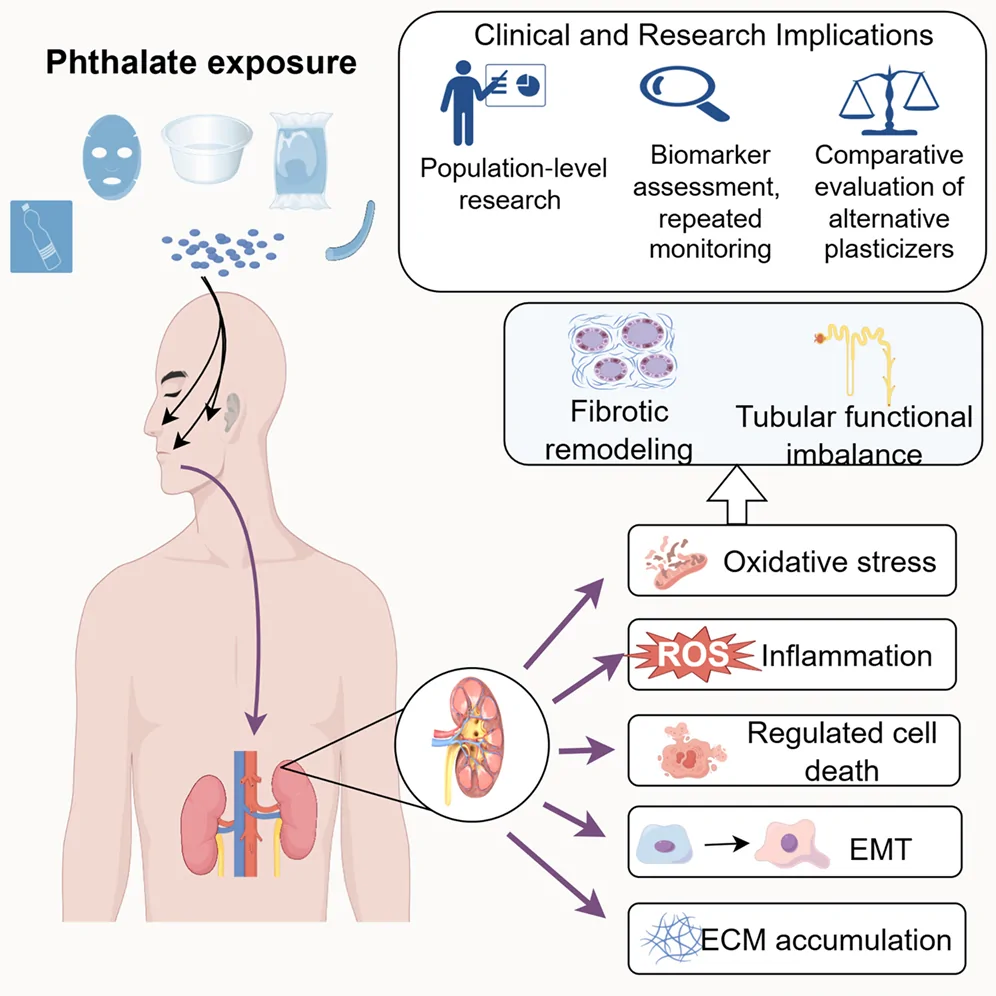

## Teaser

Health sciences; Medicine; Nephrology; Public health

## Introduction

Chronic kidney disease (CKD) is defined as abnormalities of kidney structure or function that persist for more than 3 months, commonly reflected by reduced estimated glomerular filtration rate (eGFR) and/or increased albuminuria.^1^^,^^2^ Over recent decades, CKD has shown an increasing global prevalence driven by population aging, the rising burden of metabolic disorders, and the incomplete control of traditional risk factors.^1^^,^^3^^,^^4^^,^^5^ Despite the establishment of clinical strategies for early detection and management—such as identifying CKD through routine screening, addressing major etiologies (e.g., diabetes and hypertension), optimizing blood pressure and proteinuria control, and implementing renoprotective therapies—CKD management remains challenging in real-world settings.^1^^,^^3^^,^^6^^,^^7^^,^^8^ Many patients still experience late diagnosis, heterogeneous adherence to treatment, and a persistent risk of disease progression and long-term complications, including cardiovascular morbidit.^7^^,^^9^^,^^10^ Therefore, CKD should be approached not only as a purely clinical disease but also as a condition requiring integrated management, including risk reduction for modifiable environmental exposures. In this context, prevention strategies targeting environmental pollutants are increasingly recognized as a potential complementary avenue to mitigate CKD incidence and progression.^11^^,^^12^

Phthalates are synthetic plasticizers used to enhance the flexibility, durability, and transparency of polyvinyl chloride (PVC) and other polymers, raising concern due to their ubiquity, potential for bioaccumulation, and endocrine-disrupting properties.^13^^,^^14^ Because phthalates are not chemically bound to plastics, they can readily leach into environmental media and biological fluids, leading to nearly universal human exposure.^13^^,^^15^^,^^16^ Exposure occurs via multiple pathways, including ingestion of contaminated food or dust particles, inhalation of airborne phthalate compounds, and dermal absorption from consumer products; in addition, medical-related exposure can occur through contact with plastic materials used in healthcare settings.^13^^,^^14^^,^^15^^,^^16^^,^^17^^,^^18^ Owing to their endocrine-disrupting and multi-organ toxicity potential, phthalates have been associated with a range of adverse health outcomes, including reproductive and developmental disorders, metabolic dysfunction and obesity-related phenotypes, asthma and allergic diseases, increased cardiovascular risk, impaired immune regulation, and potential carcinogenicity involving hormone-related tissues.^13^^,^^14^^,^^19^ Importantly, accumulating evidence suggests a close potential link between phthalate exposure and CKD. Epidemiological studies have reported associations between urinary phthalate metabolites and early kidney-injury indicators, such as albuminuria, and kidney-function measures; in some longitudinal studies, these metabolites were also associated with steeper declines in eGFR or increases in albuminuria.^11^^,^^12^^,^^20^ Building on this rationale, the present review summarizes the epidemiological evidence supporting associations between phthalates and CKD, outlines plausible underlying mechanisms—including oxidative stress, inflammation, apoptosis, and renal tubular injury—and proposes public health and clinical management measures aimed at reducing phthalate exposure to improve CKD prevention and control, particularly in high-risk populations.

## Literature search and review scope

This article is a structured narrative review rather than a systematic review or meta-analysis. We aimed to synthesize peer-reviewed evidence on phthalate exposure and kidney-related outcomes, including epidemiological studies of kidney function and injury markers, longitudinal renal outcomes, evidence in populations with distinctive exposure contexts, and experimental studies of biological plausibility. Literature retrieval was conducted in PubMed, Web of Science, and Embase for English-language articles published up to August 31, 2025. Search terms combined controlled vocabulary and free-text terms related to phthalates and plasticizers (e.g., “phthalate,” “DEHP,” “mono-(2-ethylhexyl) phthalate [MEHP],” and “plasticizer”) with kidney-related terms (e.g., “chronic kidney disease,” “kidney,” “renal,” “eGFR,” “albuminuria,” “nephrotoxicity,” and “kidney injury”). Reference lists of relevant reviews and eligible original articles were also screened to identify additional studies. We prioritized original human studies reporting phthalate-related exposure measures and kidney-related outcomes, together with experimental studies that examined kidney-specific or clearly kidney-relevant endpoints. Recent comprehensive reviews were used to contextualize, rather than replace, primary evidence. Studies were not selected to provide an exhaustive quantitative estimate of association, and no formal risk-of-bias assessment was undertaken. A quantitative meta-analysis was not performed because of substantial heterogeneity in study populations, phthalate compounds and mixtures, exposure matrices and dilution correction approaches, outcome definitions, analytic models, follow-up periods, and experimental systems. Accordingly, the evidence is narratively synthesized by outcome category and evidence type, with particular attention to whether a study assesses a cross-sectional renal marker, a longitudinal renal outcome, a clinical event, or experimental biological plausibility.

## Observational evidence on phthalate exposure, kidney-related markers, and related clinical outcomes

This section synthesizes observational evidence by outcome category and population: cross-sectional kidney function or kidney injury markers, longitudinal renal-marker outcomes, mortality or cardiovascular outcomes, and special exposure contexts. Most available studies assessed one-time urinary biomarkers or cross-sectional clinical measures; therefore, their findings are described as associations and are not interpreted as evidence of CKD onset or progression unless a longitudinal renal outcome was explicitly evaluated (Table 1).

**Table 1:** Observational human evidence on phthalate exposure and kidney-related outcomes

Study (Year)	Design population	Exposure assessment	Repeated samples	Outcome definition	Effect estimate	Major covariates	Key finding	Principal limitations
**A. Cross-sectional kidney-function or kidney-injury markers**								

Kang et al.^21^	cross-sectional NHANES, 2005–2016; *n* = 9,008.	spot urine; 10 phthalates; creatinine-adjusted.	1 spot urine	single eGFR and spot ACR	per ln-unit: MBP-eGFR: β = −1.32 (95% CI −1.71 to −0.94); MBzP-eGFR: β = −1.10 (−1.48 to −0.73); MBP-ACR: β = 0.04 (0.01–0.06); MBzP-ACR: β = 0.03 (0.00–0.06).	cycle, demographics, SES, lifestyle, BMI, urinary creatinine; eGFR in novel model.	MBP/MBzP inversely associated with eGFR and positively with ACR;	cross-sectional; one short-half-life measure; reverse causation;
Chen et al.^22^	community study, northeastern Taiwan, 2017–2019; *n* = 887; urine subset *n* = 120; CKD subgroup *n* = 38.	first-morning urine; 17 xenoestrogens; UPLC-MS/MS; creatinine-corrected; questionnaire score.	1 first-morning urine; urinary subset *n* = 120	eGFR, UACR and source CKD definition	high score-eGFR: β = −4.226 (SE 1.830), *p* = 0.021; MEHP-high score OR = 3.037 (95% CI 1.274–7.241); EP-UACR β = 1.934 (0.135–3.733); CKD subgroup MEHP-eGFR β = −2.465 (SE 0.794).	age, sex, BMI, BP, diabetes, eGFR, and outcome-specific clinical factors.	exposure score-eGFR, MEHP-score, EP-UACR and CKD-subgroup associations; not incident CKD evidence.	cross-sectional; one urine; small urinary subset; questionnaire/exposure misclassification.
Li et al.^23^	baseline Taiwan Biobank analysis, 2008–2015; *n* = 1,153.	morning urine; 10 phthalates + melamine; LC-MS/MS; creatinine-adjusted; estimated ADI.	1 morning urine	single log ACR; microalbuminuria threshold	WQS (7 chemicals) β = 0.144 (SE 0.046), *p* = 0.002; melamine weight = 0.573, DEHP = 0.133. high/high vs. low/low β = 0.077 (95% CI 0.015–0.139)	age, sex, BMI; interaction models also hypertension and diabetes.	melamine-phthalate WQS and high/high melamine-DEHP group associated with higher ACR; not DEHP-specific causality.	cross-sectional; one exposure measure; ADI/model assumptions; mixture attribution limited.
Lee et al.^24^	cross-sectional Korean study; women aged 20–49 years, *n* = 438; higher-BP subgroup *n* = 98.	one blood/urine sample; phthalates and other chemicals; HPLC-ESI-MS/MS; creatinine-corrected.	1 blood/urine sample	single ACR; albuminuria threshold ≥30 mg/g	most single-chemical ACR associations attenuated in the higher-BP group; 16-chemical WQS β = 0.09 (95% CI 0.03–0.16)	age, BMI, glucose, BP, region, education, parity, urinary cotinine.	WQS positive in both BP strata; most single-chemical associations attenuated at higher BP; no consistent hypertension modification.	cross-sectional; one-time measures; small higher-BP subgroup
Mu et al.^25^	NHANES 2015–2018; *n* = 1,199 with CKD or related conditions; CKD *n* = 536.	single serum panel of 28 endocrine disruptors; no repeats.	1 serum/urine assessment	single ACR; primary outcome was ACR quantiles	ECP: ACR Q10 β = 0.026 (95% CI 0.019–0.033), Q40 0.027 (0.009–0.046). grouped non-CKD vs. CKD association differed (P difference = 0.002; CIs NR).	demographics, SES, lifestyle, diet, BMI, dyslipidemia, hypertension, diabetes, hyperuricemia.	ECP associated with selected ACR quantiles; no progression inference.	cross-sectional; single assessment; reverse causation/residual confounding; multiple comparisons.
Lee et al.^26^	Korean CKD study, 2011–2020; *n* = 200; temporal trends and contemporaneous eGFR.	urinary DEP, BBzP, DEHP metabolites and DEHTP metabolite	NR	contemporaneous eGFR by CKD-severity subgroup	DEP, BBzP and DEHP metabolites decreased over time; DEHTP metabolite increased. DEHP metabolites are inversely associated with eGFR overall; DEHP/BBzP associations seen in eGFR 30–59 subgroup.	NR.	DEHP/BBzP inversely associated with eGFR in selected analyses; DEHTP increased over time	CKD severity and urinary creatinine/specific gravity complicate interpretation; other limitations NR.
Tsai et al.^27^	Taiwan mother-infant cohort; children *n* = 552 (319 boys, 233 girls; mean age ∼4 years).	child spot urine + archived maternal urine; melamine and 11 phthalates; LC-ESI-MS/MS; estimated ADI.	1 child spot + archived maternal urine	child log ACR and log NAG	boys: high melamine-ACR β = 0.259 (SE 0.085; FDR *p* = 0.018); high DEHP/DEP/DiBP-NAG β = 0.165/0.152/0.176 (SE 0.052; FDR *p* = 0.006/0.008/0.006). joint score 4 vs. 0: ACR β = 0.334 (SE 0.139), NAG β = 0.296 (SE 0.085).	age, BMI, area, maternal exposure; FDR correction.	in boys, melamine related to ACR and DEHP/DEP/DiBP to NAG; joint score related to ACR/NAG; girls null after FDR, low power.	single child sample; ACR/NAG only; transient markers; multiple comparisons.
Malits et al.^28^	baseline CKiD analysis; children with CKD aged 1–17 years; *n* = 538.	baseline urine; BPA, phthalic acid and phthalate groups; creatinine addressed dilution; no repeats.	1 baseline urine	baseline eGFR, UPCR and BP z-scores	LMW phthalates-eGFR +3.46 mL/min/1.73 m^2^ per log-unit (95% CI 1.85–5.07); UPCR −9.53% (−14.21 to −4.21).	demographics, kidney disease, birth factors, BMI, ACEi/ARB, BP, urinary creatinine/cotinine.	LMW phthalates are associated with higher eGFR and lower UPCR; directions mixed; no clear renal benefit.	cross-sectional; one measure; residual confounding/reverse causation; no progression inference.

**B. Repeated-measure or longitudinal renal-marker outcomes**								

Chen et al.^29^	prospective/retrospective Taiwan community cohort, 2013–2022; ≥3 visits; *n* = 887; urine subset *n* = 120.	visit-2 morning urine; 17 EDC metabolites; UPLC-MS/MS; creatinine-expressed.	visit-2 urine; ≥3 visits; repeated outcomes	eGFR/UACR slopes, >15% eGFR decline and >20% UACR increase	MEHP-UACR slope β = 0.206 (SE 0.086), *p* = 0.025; >15% eGFR decline OR = 1.674 (95% CI 1.158–2.421); >20% UACR increase HR = 2.190 (1.083–4.430).	age, sex, exposure score, diabetes, EDCs and baseline renal/metabolic factors.	MEHP associated with steeper UACR slope, >15% eGFR decline and >20% UACR increase; surrogate-marker evidence.	small urine subset; one exposure measure; reverse causation/residual confounding.
Charytan et al.^30^	CRIC pilot; adults with CKD; *n* = 40; years 1, 3 and 5.	24-h urine at years 1/3/5; repeated phthalate/chemical metabolites; raw and creatinine-normalized analyses.	24-h urine at years 1, 3 and 5	eGFR, UPCR, kidney failure/≥50% decline; death and CVD outcomes also assessed.	mCPP-eGFR −5.269 mL/min/1.73 m^2^/year (95% CI −9.300 to −1.237), *p* = 0.012. phthalate class above vs. below median and kidney failure/≥50% eGFR decline HR = 1.12 (0.86–1.47), NS.	time-updated/univariate models; conventional covariate adjustment limited.	mCPP associated with adverse eGFR trajectory; phthalate-class kidney-event association NS.	very small selected pilot; low power; incomplete covariates; urinary handling/reverse causation.
Jacobson et al.^31^	prospective US-Canada CKiD cohort; children with CKD; *n* = 618; 2,469 visits; up to 5 years.	serial urine; BPA, PA and 21 phthalates; HPLC-ESI-MS/MS; creatinine time-dependent.	serial urine; 2,469 visits	clinical eGFR/UPCR/BP; KIM-1, NGAL, 8-OHdG and F2-isoprostane.	PA-eGFR β = −0.38 per 1-SD ln exposure (95% CI −0.75 to −0.01). DOP-NGAL β = 0.13 (0.05–0.21); DOP-KIM-1 β = 0.30 (0.21–0.40); DEHP-NGAL β = 0.11 (0.04–0.19); DEHP-KIM-1 β = 0.13 (0.08–0.19).	demographics, kidney status, birth factors, ACEi/ARB, BMI, creatinine and BP.	PA associated with lower eGFR; DOP/DEHP groups associated with NGAL/KIM-1; clinical eGFR/UPCR mostly null.	spot urine for nonpersistent chemicals; sparse later visits; no ESKD inference.
Liu et al.^32^	three-season Wuhan panel; generally healthy children aged 4–6 or 11–13 years; *n* = 103; 287 paired measures.	seasonal morning urine; 10 phthalates; GC-MS/MS; creatinine and specific-gravity sensitivity analyses.	up to 3 visits; 287 paired measures	repeated eGFR; albuminuria infrequent	per one-fold increase: MEP −2.68% (95% CI −4.32 to −1.03), MBP −2.10% (−3.77 to −0.43), MBzP −2.33% (−4.59 to −0.07), MOP −6.01% (−12.00 to −0.01); WQS −4.03% (−6.58 to −1.47).	age, sex, BMI, passive smoking, diet/activity, education, season, urinary creatinine.	MEP/MBP/MBzP/MOP and WQS associated with lower eGFR over short follow-up; not long-term CKD evidence.	short follow-up; one spot sample per visit; limited exposure-source data; reverse causation.

**C. Mortality, cardiovascular disease, and other non-renal clinical outcomes**								

Tao et al.^33^	NHANES 2005–2018 survey analysis with NDI linkage; adults *n* = 9,605; eGFR<60 subgroup *n* = 517.	one spot urine; 11 phthalates; creatinine-adjusted; no repeats.	1 spot urine	all-cause mortality; baseline eGFR strata	eGFR<60: total DEHP HR = 1.12 (95% CI 1.01–1.25); 11-metabolite WQS HR = 1.47 (1.18–1.84).	demographics, SES, lifestyle, eGFR, metabolic syndrome, hypertension.	in eGFR<60 subgroup, total DEHP and WQS associated with mortality; survey analysis with prospective linkage, not exposure cohort.	one short-half-life measure; residual confounding; baseline eGFR cannot establish CKD chronicity.
Chou et al.^34^	Taiwan cross-sectional case-control; urothelial cancer cases *n* = 224; CKD controls *n* = 272.	one enrollment urine; 7 phthalates by LC-MS; creatinine-standardized.	1 enrollment urine	urothelial cancer versus CKD controls; non-renal cancer outcome.	MEHHP per log-unit OR = 1.42 (95% CI 1.21–1.68), *p* < 0.001; 17.6–60.6 vs. < 17.5 ng/g OR = 1.52 (1.21–2.36); >60.6 OR = 2.51 (1.98–4.35).	age, GFR, traditional medicine, occupational toxins, NSAID use.	MEHHP associated with urothelial cancer; not CKD onset/progression evidence.	cross-sectional; one urine; selection, confounding and reverse causation concerns.
Liang and Li^35^	NHANES 2005–2018 cross-sectional analysis; participants with CKD, *n* = 834.	one spot urine; 10 phthalates; creatinine-adjusted; no repeats.	1 spot urine	prevalent self-reported CVD; not incident CVD or CKD progression.	highest vs. lowest tertile: MiBP OR = 1.86 (95% CI 1.08–3.18), MCNP 1.85 (1.18–2.91), MBP 1.68 (1.02–2.76), MECPP 2.22 (1.28–3.86). WQS per quartile OR = 1.44 (1.04–1.99).	demographics, SES, BMI, smoking, hypertension, diabetes, cholesterol, eGFR.	several metabolites and WQS associated with prevalent CVD; mixture index reflects joint exposure, not single-chemical causality.	cross-sectional; self-reported CVD; one urine; residual confounding/reverse causation.
Chen et al.^36^	NHANES 2003–2018 cross-sectional analysis; participants with CKD, *n* = 2,227.	one urine sample; 8 phthalates; dilution-adjusted; no repeats.	1 urine sample	prevalent self-reported CVD; CKD persistence not established.	single metabolites: MBP OR = 1.26 (95% CI 1.09–1.45), MBzP 1.25 (1.08–1.43), MEHHP 1.13 (1.01–1.27), MEOHP 1.20 (1.07–1.35), MiBP 1.22 (1.06–1.40), MECPP 1.18 (1.05–1.33). WQS OR = 1.19 (1.00–1.41); qgcomp OR = 1.23 (1.08–1.40).	demographics, SES, BMI, hypertension, diabetes, smoking, alcohol, lipids; FDR.	several metabolites, WQS and qgcomp associated with prevalent CVD; mixture methods do not prove single-chemical causality.	cross-sectional; self-reported CVD; one urine; incomplete diet/medication/dialysis data.
Zhang et al.^37^	NHANES survey analysis with NDI linkage; adults with diabetes, *n* = 3,453; DKD baseline subgroup.	baseline urine; phenols and phthalates; no repeats.	1 baseline urine	all-cause mortality; baseline DKD definition.	DKD per log-unit: MEHHP HR = 1.24 (95% CI 1.08–1.42), MEOHP 1.27 (1.10–1.47), MECPP 1.43 (1.19–1.71). highest vs. lowest tertile: MEHHP 1.76 (1.07–2.89), MEOHP 1.66 (1.04–2.63), MECPP 2.34 (1.38–3.96)	demographics, diabetes duration, HbA1c, BP, LDL-C.	MEHHP, MEOHP and MECPP associated with mortality in baseline DKD subgroup; linkage analysis, not CKD progression.	one urine; DKD chronicity and reverse causation; residual confounding.

**D. Special exposure-context evidence**								

Dine et al.^38^	hemodialysis; *n* = 11; pharmacokinetic study.	4-h session; venous-blood DEHP by HPLC; dialyzer/tubing exposure; AUC model.	1 4-h dialysis session	intradialytic exposure and modeled DEHP retention; no clinical kidney outcome.	blood DEHP increased across the apparatus; estimated retention varied widely and could reach toxic levels with repeated sessions; exact estimates/CI/P: NR.	NR	venous-blood DEHP increased; modeled retained amount showed high interpatient variability; repeated exposure may reach a toxicological level.	model-based inference; small study; no CKD progression outcome.
Mettang et al.^39^	hemodialysis; *n* = 21; pre/post session study.	pre/post serum DEHP, MEHP, 2-EH and PA; GC/SIM-MS.	pre/post dialysis session	immediate exposure; uremic pruritus	post-dialysis DEHP/MEHP/2-ethylhexanol increased; phthalic acid decreased (0.122 ± 0.078 vs. 0.194 ± 0.101 μg/μL, *p* = 0.00068). no concentration-pruritus association.	NR	post-dialysis DEHP/MEHP/2-EH increased and PA decreased; no metabolite-pruritus correlation.	small short-term study; exposure context; no CKD progression outcome.
Sachs-Strømmen et al.^40^	kidney-transplanted children; *n* = 52; cross-sectional.	one exposure assessment; BPA intake and propylparaben HQ.	1 exposure assessment	estimated intake/risk screen; contemporaneous GFR.	MEP-GFR Spearman r = 0.33, *p* = 0.02. all had BPA EDI above TDI; 13% had propylparaben HQ > 1. HQ > 1 is a screening exceedance, not an observed clinical effect.	NR.	BPA intakes exceeded selected TDI; propylparaben HQ > 1 in 13%; exploratory MEP-GFR correlation.	HQ > 1 is screening exceedance, not clinical harm; small cross-sectional study.

ACR, albumin-to-creatinine ratio; ADI, average daily intake; AUC, area under the curve; BPA, bisphenol A; BP, blood pressure; BP-3, benzophenone-3; BMI, body mass index; CI, confidence interval; CKD, chronic kidney disease; CRIC, chronic renal insufficiency cohort; CVD, cardiovascular disease; DEHP, di-(2-ethylhexyl) phthalate; DEHTP, di-(2-ethylhexyl) terephthalate; DEP, diethyl phthalate; DKD, diabetic kidney disease; ECP, ethyl phthalate-related metabolite label used in the source study; EDC, endocrine-disrupting chemical; EDI, estimated daily intake; ESKD, end-stage kidney disease; ESI, electrospray ionization; F2-isoprostane, 8-iso-prostaglandin F2α; FDR, false-discovery rate; GC, gas chromatography; GFR, glomerular filtration rate; HPLC, high-performance liquid chromatography; HQ, hazard quotient; HR, hazard ratio; KIM-1, kidney injury molecule-1; LC, liquid chromatography; LDL-C, low-density lipoprotein cholesterol; LMW, low molecular weight; MBP, mono-*n*-butyl phthalate; MBzP, monobenzyl phthalate; MCNP, mono-carboxynonyl phthalate; MECPP, mono-(2-ethyl-5-carboxypentyl) phthalate; MEHHP, mono-(2-ethyl-5-hydroxyhexyl) phthalate; MEHP, mono-(2-ethylhexyl) phthalate; MEOHP, mono-(2-ethyl-5-oxohexyl) phthalate; MEP, monoethyl phthalate; MECP/“MECP phthalate,” the source study’s label for ECP; MCPP, mono-(3-carboxypropyl) phthalate; MOP, mono-*n*-octyl phthalate; MS, mass spectrometry; NDI, National Death Index; NGAL, neutrophil gelatinase-associated lipocalin; NAG, N-acetyl-β-D-glucosaminidase; NR, not reported; NSAID, nonsteroidal anti-inflammatory drug; NS, not statistically significant; OR, odds ratio; PA, phthalic acid; Q10/Q20/Q30/Q40, the 10th/20th/30th/40th percentiles; SE, standard error; SES, socioeconomic status; SIM, selected-ion monitoring; TDI, tolerable daily intake; UACR, urine albumin-to-creatinine ratio; UPCR, urine protein-to-creatinine ratio; UPLC, ultra-performance liquid chromatography; US, United States; WQS, weighted quantile sum; 2-EH, 2-ethylhexanol; 8-OHdG, 8-hydroxy-2′-deoxyguanosine; α-SMA, alpha-smooth muscle actin. The abbreviation MECP/MECP phthalate is retained as reported in the source study and should not be equated with MECPP. For mixture analyses, WQS weights describe relative contributions within the modeled mixture and do not establish independent causal effects. For study-specific symbols, β denotes a regression coefficient and P denotes the corresponding *p* value.

### Phthalate exposure and kidney function or early kidney-injury markers in the general population

In cross-sectional studies of the general population, several urinary phthalate metabolites have been associated with kidney function or kidney injury markers. In National Health and Nutrition Examination Survey (NHANES) 2005–2016 (*n* = 9,008), covariate-adjusted creatinine standardization showed inverse associations of mono-n-butyl phthalate (MBP) and mono-benzyl phthalate (MBzP) with eGFR: each ln-unit increase in MBP and MBzP was associated with decreases of 1.32 and 1.10 mL/min/1.73 m^2^, respectively. Both metabolites were also positively associated with spot urinary ACR. In weighted quantile sum (WQS) analyses, MBzP was the leading contributor to the inverse eGFR and positive ACR mixture associations.^21^ Because WQS estimates the association of the overall exposure mixture, the component weight for MBzP does not establish an independent causal effect of MBzP or of any other mixture component. As these measurements were obtained at one survey visit, they represent cross-sectional associations with renal markers rather than incident CKD. A community-based study in northeastern Taiwan (*n* = 887) reported that a higher questionnaire-derived xenoestrogen exposure score was associated with lower eGFR. In the urinary-analysis subset (*n* = 120), MEHP was associated with a higher exposure-score category, whereas ethylparaben was associated with higher urine albumin-to-creatinine (UACR). In the small CKD subgroup (*n* = 38), MEHP was inversely associated with eGFR^22^; in a separate longitudinal analysis of this population, urinary MEHP measured at the second visit was associated with a steeper UACR slope (β = 0.206, *p* = 0.025), a greater than 15% eGFR decline (odds ratio [OR] = 1.674, 95% confidence interval [CI] 1.158–2.421), and a greater than 20% increase in UACR (hazard ratio [HR] = 2.190, 95% CI 1.083–4.430).^29^

In the Taiwan Biobank study (*n* = 1,153), the WQS index for melamine and six phthalate estimated daily intakes was associated with higher single-timepoint ACR (β = 0.144, *p* = 0.002). Melamine contributed the largest WQS weight (0.573), whereas di-(2-ethylhexyl) phthalate (DEHP) contributed 0.133. The high-melamine/high-DEHP group had higher ACR than the low-melamine/low-DEHP group; this finding should therefore be interpreted as an association with the joint melamine-DEHP exposure pattern rather than as evidence of an independent DEHP effect.^23^ The WQS weights quantify the relative contribution of components within the modeled mixture and do not demonstrate independent causal effects or a biological interaction for melamine, DEHP, or any other component. Blood-pressure context may influence some cross-sectional findings, although the direction is not uniform across analytical approaches. In Korean women of reproductive age (*n* = 438), most single-organic-chemical associations with ACR observed in the normal/elevated blood-pressure group attenuated in the prehypertension/hypertension group; mono-(2-ethyl-5-hydroxyhexyl) phthalate (MEHHP) and BP-1 were exceptions. Conversely, the WQS mixture index was positively associated with ACR in the overall sample and in both blood-pressure strata. Given the small prehypertension/hypertension stratum and the difference between single-chemical and mixture models, these findings do not establish that hypertension consistently strengthens or weakens the MEHHP-ACR association.^24^ A further NHANES analysis of 1,199 adults reported cross-sectional associations between ACR quantiles and MECP phthalate (ECP). The grouped analysis indicated an association in the non-CKD group but not in the CKD group.^25^ Overall, these studies support heterogeneous cross-sectional associations with kidney-related markers, but single-spot exposure assessment and potential reverse causation limit causal interpretation.

### Phthalate exposure in CKD populations and associations with longitudinal markers, mortality, and cardiovascular outcomes

Evidence specifically in people with CKD is limited and requires careful interpretation because kidney dysfunction can alter urinary metabolite excretion. In a chronic renal insufficiency cohort (CRIC) pilot study (*n* = 40), time-updated analyses identified a significant association only for mono-(3-carboxypropyl) phthalate (mCPP), for which a one-log10-unit increase was associated with a faster eGFR decline (−5.269 mL/min/1.73 m^2^/year, 95% CI −9.300 to −1.237). Associations for the phthalate class with kidney failure or a 50% eGFR decline were not significant.^30^ A Korean study of 200 CKD patients reported calendar-time decreases in several conventional phthalate metabolites and an increase in a di(2-ethylhexyl) terephthalate (DEHTP) metabolite. In cross-sectional analyses, negative associations of DEHP and BBzP exposure with eGFR were observed in the moderate-dysfunction subgroup (eGFR 30–59 mL/min/1.73 m^2^), but findings were not consistent across CKD-severity strata.^26^

Several studies evaluated non-renal clinical outcomes rather than CKD progression. Tao et al. used baseline NHANES urine measurements (2005–2018) linked to the National Death Index through 2019; this is a survey analysis with prospective mortality linkage, not a conventional prospective exposure cohort. In participants with baseline eGFR <60 mL/min/1.73 m^2^, total DEHP metabolites were associated with all-cause mortality (HR = 1.12, 95% CI 1.01–1.25), and the 11-metabolite WQS score was also associated with mortality (HR = 1.47, 95% CI 1.18–1.84).^33^ A Taiwanese case-control study found that urinary MEHHP was associated with prevalent urothelial cancer among CKD controls and cases.^34^

Other NHANES analyses assessed prevalent self-reported cardiovascular disease (CVD) among participants classified as CKD. In one study (*n* = 834), higher urinary phthalate-metabolite tertiles and a WQS index were associated with prevalent CVD.^35^ A separate study of 2,227 NHANES participants classified as CKD found positive cross-sectional associations for several individual metabolites and for phthalate-mixture indices with prevalent self-reported CVD.^36^ A prospective NHANES-linked mortality study in 3,453 adults with diabetes evaluated all-cause mortality through 2019. In the subgroup meeting a baseline diabetic kidney disease definition based on single ACR/eGFR measurements, MEHHP, mono-(2-ethyl-5-oxohexyl) phthalate (MEOHP), and mono-(2-ethyl-5-carboxypentyl) phthalate (MECPP) were associated with all-cause mortality (HRs 1.24, 1.27, and 1.43, respectively, per log-unit increase).^37^

### Children, dialysis patients, and transplant recipients: Susceptible populations and special exposure contexts

Studies in children with CKD provide repeated-measure evidence for tubular injury and oxidative-stress biomarkers, but clinical renal outcomes have been less consistent. In the US-Canada CKD in Children (CKiD) cohort (*n* = 618; 2,469 visits), repeated bisphenol A (BPA) and phthalate-group exposures were associated with higher urinary kidney injury molecule-1 (KIM-1), neutrophil gelatinase-associated lipocalin (NGAL), 8-hydroxy-2′-deoxyguanosine (8-OHdG), and F2-isoprostane. For example, di-*n*-octyl phthalate metabolites were associated with higher NGAL and KIM-1; however, BPA and phthalate groups were not associated with eGFR or proteinuria, except for an inverse association between phthalic acid and eGFR.^31^ A three-season Chinese panel study of 103 generally healthy children (287 paired urine-blood measurements) found that each 1-fold increase in monoethyl phthalate (MEP), MBP, MBzP, and mono-n-octyl phthalate (MOP) was associated with 2.68%, 2.10%, 2.33%, and 6.01% lower eGFR, respectively. The negative-direction WQS mixture was associated with a 4.03% lower eGFR. These were short-term repeated-measure associations; they do not represent a long-term eGFR slope or clinical CKD progression.^32^ In a Taiwanese mother-infant cohort (*n* = 552), analyses were conducted separately for boys and girls. Among boys, high estimated melamine intake was associated with higher ACR, whereas high estimated intakes of DEHP, diethyl phthalate (DEP), and DiBP were associated with higher N-acetyl-β-D-glucosaminidase (NAG). Boys with a higher cumulative multiple-exposure score also had higher ACR and NAG. In contrast, no individual high-exposure or cumulative-score association with ACR or NAG remained statistically significant among girls after false-discovery-rate correction. However, the number of girls in the highest cumulative score category was small (*n* = 16); therefore, the absence of statistical significance in girls should not be interpreted as evidence of no effect or as conclusive evidence of a sex difference.^27^ These findings should therefore be interpreted as sex-stratified associations rather than confirmation of a sex-by-exposure interaction or greater male susceptibility. In a separate CKiD baseline analysis (*n* = 538), low-molecular-weight phthalates were associated with higher eGFR and lower urine protein-to-creatinine ratio (UPCR), findings that may reflect hyperfiltration and/or altered urinary handling rather than renal benefit.^28^

In dialysis settings, plasticized tubing and dialyzers can provide a distinct medical device exposure source.^17^^,^^18^ A pharmacokinetic study of 11 hemodialysis patients showed intradialytic increases in blood DEHP and substantial interindividual variation in estimated DEHP retention.^38^ Another pre/post-dialysis study of 21 patients showed increased post-dialysis DEHP, MEHP, and 2-ethylhexanol concentrations, but no immediate association between DEHP-related measures and uremic-pruritus severity.^39^ These studies demonstrate special exposure contexts rather than renal-progression outcomes. In Norwegian kidney-transplanted children (*n* = 52), a cross-sectional exposure and risk-assessment study reported that all participants had estimated BPA intakes above the selected tolerable daily intake, and 13% had propylparaben hazard quotients above 1. A hazard quotient above 1 indicates a potential risk-screening exceedance, not an observed adverse clinical effect or a comparison with an unexposed reference group. The study reported an exploratory positive correlation between MEP and contemporaneous GFR but no graft-failure, CKD-progression, or kidney-injury outcome.^40^ Taken together, these studies indicate that children, dialysis patients, and transplant recipients may experience distinct exposure patterns and biomarker associations; larger studies with repeated exposure and clinical-outcome assessment are required before causal conclusions can be made.

### Interpreting urinary phthalate biomarkers in the setting of kidney dysfunction

A central interpretive challenge in this field is the bidirectional relationship between kidney function and measured urinary phthalate-metabolite concentrations. Urinary biomarkers are valuable indicators of recent elimination, but they should not be interpreted as direct measures of external exposure, stable whole-body burden, or target-tissue dose. An uncorrected urinary concentration (for example, ng/mL) is affected by recent intake, sampling time, metabolism, urine volume and renal excretion. A creatinine-corrected concentration (for example, μg/g creatinine) can reduce variation related to urine dilution, but creatinine excretion itself is influenced by kidney function, muscle mass, diet, age, sex and clinical condition. Estimated daily intake (EDI) is a model-derived back-calculation from urinary biomarkers and depends on assumptions about urinary excretion fractions, metabolism and physiological parameters. Plasma or serum concentration provides a complementary circulating biomarker, but it is also shaped by distribution, protein binding, metabolism and renal clearance. External exposure, internal body burden, urinary concentration, creatinine-corrected concentration, EDI and plasma concentration are therefore related but non-interchangeable constructs.

This distinction is particularly important in CKD. Phthalate exposure may plausibly contribute to kidney injury; however, established kidney dysfunction may also affect measured urinary metabolite concentrations through reduced filtration, changes in tubular secretion or reabsorption, altered urine volume and altered creatinine excretion. In addition, dietary changes, medication use, hydration, muscle mass and medical-device exposure may influence exposure opportunity, biomarker concentration, or both. Accordingly, a lower urinary concentration in advanced CKD does not necessarily indicate lower external exposure or lower internal body burden. Conversely, a higher urinary concentration may reflect recent exposure, altered renal handling, reduced tubular reabsorption, urine concentration, or treatment-related exposure. Creatinine correction and covariate-adjusted standardization can address selected sources of variation, but they cannot fully eliminate reverse causation or measurement bias when kidney function itself affects the biomarker pathway.

Future studies should prioritize repeated exposure assessment, concurrent measurement of renal function and urinary dilution markers, transparent reporting of the dilution correction strategy, and, where feasible, complementary matrices or exposure-source data. Associations based on a single urinary measurement should be interpreted cautiously as observational; longitudinal studies provide stronger temporal information but still require careful consideration of exposure misclassification and reverse causation. In CKD, dialysis and transplant populations, analyses should distinguish baseline external exposure, internal body burden, renal elimination and treatment-related exposure sources rather than inferring one construct directly from a single urinary metabolite concentration. Figure 1 summarizes this conceptual framework.

**Figure 1:**
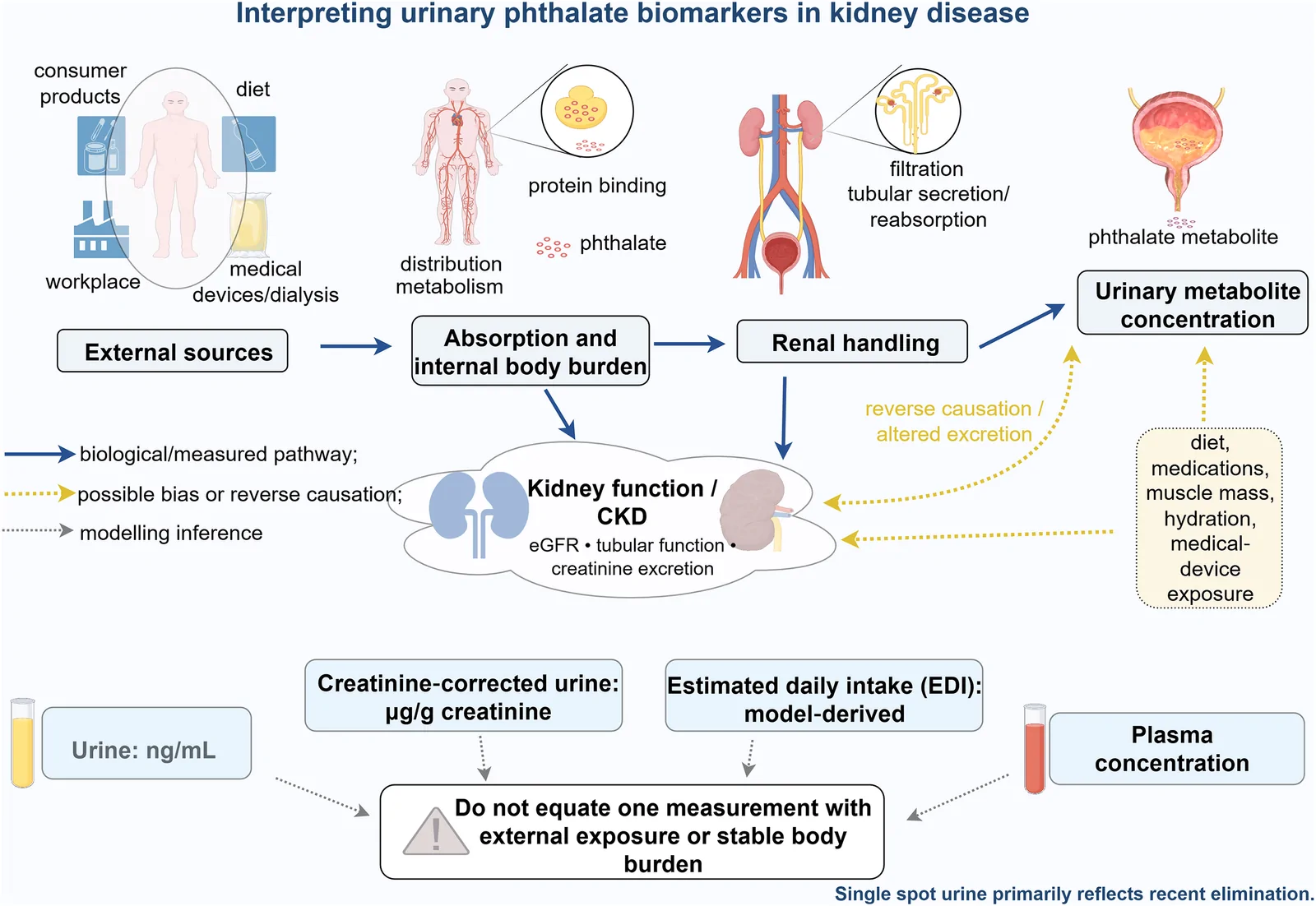
Conceptual framework for interpreting urinary phthalate biomarkers in kidney disease External sources, including diet, consumer products, workplace exposure, and medical device- or dialysis-related exposure, contribute to absorption, internal distribution, metabolism, and body burden. Renal filtration, tubular secretion or reabsorption, urine volume, and creatinine excretion may influence the measured urinary concentration of phthalate metabolites. Conversely, kidney dysfunction may alter metabolite elimination and urinary measurements, creating potential reverse causation or altered-excretion bias. Urinary concentration, creatinine-corrected concentration, estimated daily intake, and plasma concentration represent different exposure-related metrics and should not be interpreted as interchangeable measures of external exposure or stable internal body burden. Single-spot urine measurements primarily reflect recent elimination and require cautious interpretation in individuals with kidney disease.

## Potential mechanisms linking phthalates to CKD

While human studies have reported associations between phthalate exposure and kidney-related outcomes across metabolites and populations, the mechanistic basis remains incompletely resolved, and the supporting data are derived from heterogeneous study models with varying translational relevance. Recent reviews have comprehensively summarized the molecular mechanisms of phthalate- and DEHP-associated nephrotoxicity. In particular, Quasmi et al. reviewed multiple phthalates and major pathways involving oxidative stress, inflammation, mitochondrial injury, DNA damage, ferroptosis, and pharmacological interventions, whereas Liu et al. provided a DEHP-focused overview of apoptosis, autophagy, ferroptosis, oxidative stress, inflammation, lipid metabolism, and preclinical protective compounds. Rather than duplicating these established pathway inventories, the present review retains a concise mechanism-oriented synthesis while integrating kidney-focused mechanisms with contextual issues that are less emphasized in previous reviews, including environmental co-exposure that may modify susceptibility to other nephrotoxicants and potential interference with CKD-related pharmacological pathways. Figure 2 is therefore presented as a proposed evidence framework, with color-coded labels indicating the predominant evidence context, including human observational evidence, mammalian experimental evidence, non-mammalian or *in vitro* evidence, and indirect or extra-renal inference. Findings from non-mammalian models, *in vitro* systems, or extra-renal cell types require targeted renal validation before being extrapolated to human CKD pathogenesis. Although the epidemiological literature reviewed here includes multiple phthalate metabolites and selected alternative plasticizers, direct experimental evidence for renal mechanisms is predominantly derived from DEHP or its principal metabolite MEHP. Accordingly, the mechanistic pathways discussed in this section should be interpreted as a DEHP/MEHP-dominant framework rather than as class-wide mechanisms shared by all phthalates. Data on other phthalates, including dibutyl phthalate (DBP), DiNP, and butyl benzyl phthalate (BBP), remain comparatively limited, and their renal effects may differ because of compound-specific exposure sources, metabolism, tissue distribution, and toxicological potency. These mechanisms should not be extrapolated to chemically distinct alternative plasticizers without compound-specific evidence. This approach is intended to clarify the translational relevance and remaining uncertainties of the available mechanistic literature.

**Figure 2:**
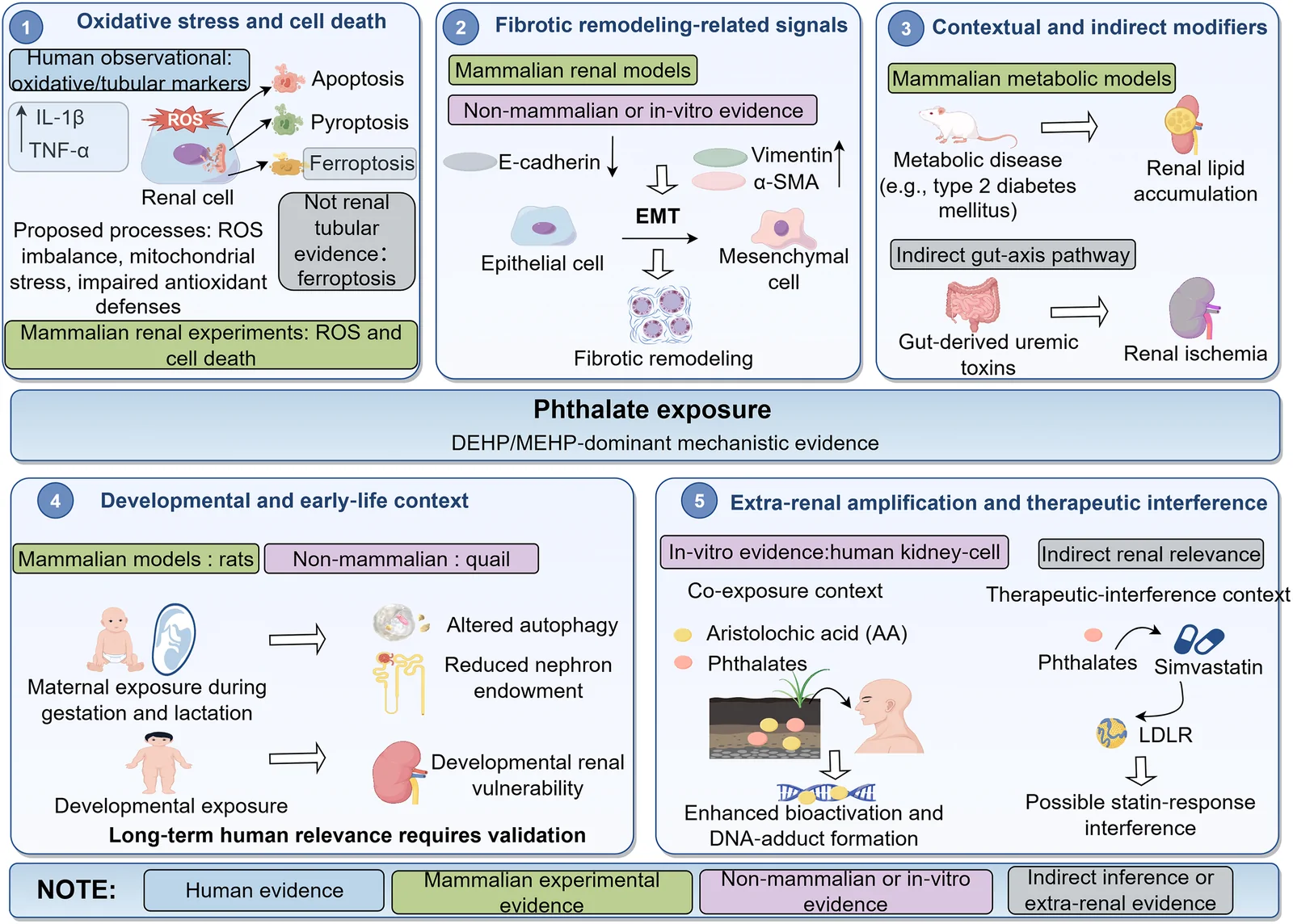
Proposed evidence framework linking phthalate exposure with kidney-related outcomes Phthalate exposure, with the mechanistic evidence reviewed here predominantly derived from DEHP and its principal metabolite MEHP, may be associated with several potentially interconnected biological processes. The mechanisms summarized in the main renal injury pathways should not be interpreted as class-wide mechanisms established for all phthalates or chemically distinct alternative plasticizers. In renal experimental systems, oxidative stress-related processes include excessive reactive oxygen species (ROS) generation, mitochondrial stress, impaired antioxidant defenses, and inflammatory signaling involving interleukin-1β (IL-1β), tumor necrosis factor-α (TNF-α), and NF-κB. These processes have been investigated in relation to regulated cell-death phenotypes, including apoptosis, pyroptosis, and ferroptosis. Fibrotic remodeling-related signals include epithelial-mesenchymal transition (EMT), characterized by decreased E-cadherin and increased α-smooth muscle actin (α-SMA) and vimentin, together with fibrotic remodeling. Contextual and indirect modifiers include metabolic disease models, renal lipid accumulation, and gut-axis pathways involving gut-derived uremic toxins and renal ischemia. Developmental and early-life contexts include maternal exposure during gestation or lactation, developmental exposure, altered autophagy, reduced nephron endowment, and developmental renal vulnerability. The extra-renal and therapeutic-interference panel summarizes environmental co-exposure findings involving selected phthalate esters, including DEP, DBP, and BBP, together with aristolochic acid (AA) in cultured human kidney cells, as well as possible DEHP-related statin-response interference investigated in extra-renal cell systems, including Huh7 hepatocytes and endothelial cells. The color-coded labels indicate the predominant evidence context: blue, human observational evidence; green, mammalian experimental evidence; purple, non-mammalian or *in vitro* evidence; and gray, indirect inference or extra-renal evidence. Human kidney-cell experiments are therefore classified as *in vitro* evidence rather than human clinical evidence, and hepatocyte findings do not constitute direct evidence of renal tubular ferroptosis. The figure presents a proposed evidence framework rather than a deterministic causal cascade; the contribution of each pathway to human CKD remains to be established through renal-specific, longitudinal, and interventional research.

### Oxidative stress as a potential upstream mechanism

In experimental renal models, DEHP and its metabolites have been associated with disruption of redox balance in kidney cells, including increased generation of reactive oxygen species (ROS) and depletion of antioxidant defenses.^41^^,^^42^ In experimental models, DEHP and selected other phthalates have been associated with reduced antioxidant capacity (e.g., decreased circulating or renal glutathione peroxidase activity) alongside enhanced oxidative damage readouts such as intensifying lipid peroxidation markers (e.g., increased 4-hydroxy-2-nonenal staining).^43^^,^^44^^,^^45^^,^^46^^,^^47^^,^^48^^,^^49^ Once ROS accumulate, they function as an upstream trigger that propagates renal inflammatory signaling.^50^ In mouse kidneys, DEHP-induced ROS accumulation activates nuclear factor kappa B (NF-κB), leading to upregulation of pro-inflammatory cytokines such as interleukin-1β (IL-1β) and tumor necrosis factor-α (TNF-α), thereby accelerating renal inflammatory responses.^51^^,^^52^ In a mouse co-exposure model, DEHP combined with polystyrene microplastics was associated with renal NF-κB activation, and N-acetylcysteine attenuated the inflammatory response.^51^ As this was a particle-chemical co-exposure experiment, the finding should be interpreted as compatible with a ROS-mediated response to combined stress rather than as evidence that DEHP alone activates the same pathway. The independent, additive, and interactive contributions of DEHP and microplastics require studies with appropriate single-exposure comparator groups. Consistent with this ROS-inflammation linkage, pharmacologic modulation by antioxidants such as quercetin can inhibit DEHP-triggered NF-κB activation in rat kidneys.^52^

Oxidative stress is further amplified by mitochondrial dysfunction, which turns redox disturbance into a self-reinforcing injury environment. DEHP exposure reduces mitochondrial membrane potential (ΔΨm), induces mitophagy, and impairs mitochondrial energetics.^53^ As mitochondrial damage progresses, mitochondrial DNA (mtDNA) is released into the cytosol, where it activates the cyclic guanosine monophosphate (GMP)-AMP synthase (cGAS)-stimulator of interferon genes (STING) signaling axis, amplifying inflammatory responses and contributing to apoptosis. This mitochondrial-to-inflammatory escalation is also reported to be reversible under targeted interventions: for example, lycopene (LYC) reverses DEHP-linked inflammatory amplification by modulating PTEN-induced kinase 1 (PINK1)/Parkin activation and suppressing cGAS-STING signaling.^53^ In a long-term exposure model, DEHP was associated with glomerular oxidative stress changes, including increased nicotinamide adenine dinucleotide phosphate (NADPH) oxidase activity, reduced antioxidant enzyme levels, and diminished peroxisome proliferator-activated receptor alpha (PPARα)-dependent suppression of NF-κB. These changes were accompanied by immune complex glomerulonephritis in the experimental model.^54^ The findings support a possible association between DEHP-related oxidative stress and glomerular injury, but they do not establish that this pathway drives progressive CKD in humans. Human observational evidence referenced further aligns with this mechanism framework: urinary phthalate-related measures correlate with systemic/tubular oxidative stress markers and tubular injury signals over time,^31^ and meta-analytical evidence across mammalian studies supports that phthalate exposure elevates renal oxidative damage markers while depleting antioxidant defenses, while noting that many animal experiments used high doses requiring cautious extrapolation.^55^

### Potential regulated cell death and renal parenchymal injury

DEHP and selected other phthalates may be associated with renal parenchymal injury through regulated cell death responses in tubular epithelial cells.^41^^,^^42^ In this context, apoptosis has been reported in experimental models of selected phthalates, including DBP, DEHP, and DiBP. These studies describe alterations in pro-survival signaling, such as the phosphatidylinositol 3-kinase (PI3K)/protein kinase B (AKT) pathway, attenuation of antioxidant defense signaling involving nuclear factor erythroid 2-related factor 2 (Nrf2)/heme oxygenase-1 (HO-1), and mitochondrial dysfunction, accompanied by changes in B-cell lymphoma-2 (Bcl-2) family proteins and caspase activation.^56^^,^^57^^,^^58^ Beyond apoptosis, ferroptosis has been proposed as a potential mechanism in DEHP-associated renal injury. Renal-specific evidence is derived predominantly from experimental models, whereas observations from non-mammalian or extra-renal cell systems provide indirect mechanistic support and require validation in mammalian renal tubular models.^45^^,^^59^^,^^60^ Separately, DEHP-related experimental studies have reported pyroptosis-associated changes in renal tissue, involving ROS-associated NOD-like receptor family pyrin domain containing 3 (NLRP3) inflammasome signaling, gasdermin D cleavage, and IL-1β/IL-18 release.^51^^,^^61^ This ROS dependence is shown by the ability of N‑acetylcysteine (NAC) or the NLRP3 inhibitor MCC950 to ameliorate pyroptosis and associated renal injury. And LYC can inhibit DEHP-induced caspase-1/NLRP3-dependent pyroptosis.^61^

### Fibrogenic remodeling through EMT and ECM accumulation

In experimental models, DEHP-related renal injury has been associated with fibrogenic remodeling, in which epithelial programs shift toward a mesenchymal/pro-fibrotic phenotype and extracellular matrix (ECM) accumulates. In this setting, epithelial-mesenchymal transition (EMT) is a central morphological and molecular feature of renal fibrosis, and DEHP-associated signaling involving AKT/NF-κB, GSK3β, and PPARα/peroxisome proliferator-activated receptor gamma (PPARγ) has been implicated in this process.^41^^,^^62^ DEHP exposure has been reported to induce EMT-like phenotypic switching in renal tubular epithelial cells, characterized by upregulated mesenchymal markers (e.g., α-smooth muscle actin and vimentin) alongside suppressed epithelial markers such as E-cadherin. This process has been linked to AKT/NF-κB-related signaling, together with regulation of glycogen synthase kinase 3 beta (GSK3β) and downregulation of PPARα/PPARγ, and the degree of EMT-associated changes can be partially modified by pharmacologic PPARγ activation (e.g., pioglitazone).^62^ *In vivo*, DEHP has enhanced fibrosis in experimental kidney injury models, consistent with the idea that EMT-associated signaling contributes to a progressive fibrotic phenotype rather than representing a purely transient response.^62^

### Metabolic dysfunction and gut axis-driven renal aggravation

DEHP-related metabolic dysfunction may modify renal susceptibility in disease contexts, while gut microbiota-kidney interactions may provide an additional framework for kidney injury and CKD-related processes.^63^^,^^64^ DEHP exposure has been reported to exacerbate renal injury in adolescent type 2 diabetes mellitus models by worsening insulin resistance and perturbing glucose/lipid metabolism, with downstream consequences including more pronounced renal lipid accumulation and tubular injury.^63^ Separately, gut-derived uremic toxins and barrier dysfunction may engage kidney-relevant signaling pathways such as aryl hydrocarbon receptor (AHR)/NF-κB, which have been associated with inflammatory and fibrotic responses in experimental or disease-related contexts.^64^^,^^65^^,^^66^^,^^67^^,^^68^^,^^69^ Disruption of the gut ecosystem may also affect sympathetic and blood-pressure-related pathways and could contribute to renal ischemic stress; however, whether these processes materially influence CKD progression in the context of phthalate exposure remains uncertain.^64^

### Early-life programming as a potential modifier of renal vulnerability

Early exposure can shape long-term renal risk by epigenetic and metabolic reprogramming.^41^^,^^70^ A parallel mechanism underlying life-course susceptibility is early epigenetic reprogramming. Maternal exposure to phthalates during gestation and lactation has been discussed under developmental origins of health and disease (DOHAD) concepts, whereby altered developmental signaling can translate into lasting changes in fibrogenic signaling tone.^41^^,^^70^ Experimental evidence includes fetal renal tubular disruption (e.g., reduced nephron endowment through impaired developmental pathways such as hedgehog signaling) and aberrant autophagy trajectories,^71^^,^^72^^,^^73^ which together may impair organelle quality control and facilitate tubular dysfunction that later manifests as chronic injury.^70^ Developmental exposure has been proposed to program persistent renal vulnerability through long-lasting endocrine-vascular and oxidative stress adaptations.^70^^,^^74^ In animal models, maternal DEHP exposure has been linked to offspring hypertension and renal oxidative damage,^74^ with mechanistic involvement of the renin-angiotensin system (RAS) in promoting angiotensin-converting enzyme (ACE) and angiotensin II type 1 receptor (AT1R) expression and maintaining vasoconstrictive and inflammatory signaling into adulthood.^70^ Such developmental programming is consistent with the notion that the kidney’s baseline resilience is altered early, making later environmental toxicant exposure more harmful.^70^ Taken together, early-life programming and metabolic dysfunction provide a plausible means by which the same exposure can yield stronger renal consequences in susceptible individuals, although the relative weight, timing, and reversibility of each component remain incompletely resolved.

### Extra-renal and contextual mechanisms: Co-exposure and therapeutic-interference hypotheses

Beyond direct renal toxicity, selected phthalates including DEP, DBP, and BBP may amplify nephrotoxicity through extra-renal pathways such as environmental co-exposure synergism and interference with standard CKD therapies. These indirect mechanisms are less frequently reviewed but may be critical in real-world exposure scenarios and clinical management contexts.

From the perspective of environmental co-exposure, phthalates may act as “potential modifiers of toxicity” in settings of widespread environmental co-contamination. In Balkan endemic nephropathy-endemic regions, higher concentrations of DEP, DBP, and BBP were detected in maize, wheat, and soil, indicating co-occurrence with aristolochic acid (AA) exposure.^75^ In cultured human kidney cells, co-exposure to phthalates and aristolochic acid I (AA-I) increased AA-DNA adduct formation, and this response was associated with cytochrome P450 1A1 (CYP1A1)/cytochrome P450 1B1 (CYP1B1)-related bioactivation.^76^ Because these findings derive from environmental correlation and cell-based experiments, they should be interpreted as hypothesis-generating evidence of a possible co-exposure effect rather than proof of synergism or an independent DEHP effect.

In terms of therapeutic interference, preclinical and observational findings raise the possibility that phthalate exposure may influence pathways relevant to CKD treatment response. In Huh7 hepatocytes, DEHP and its metabolites downregulated the low-density lipoprotein receptor (LDLR) via the NADPH oxidase (NOX)-ROS-transient receptor potential ankyrin 1 (TRPA1) pathway, which subsequently activated PPARγ proprotein convertase subtilisin/kexin type 9 (PCSK9) and the liver X receptor α (LXRα)-inducible degrader of LDLR (IDOL) signaling. This promotes LDLR degradation and reduces simvastatin-associated LDLR-mediated LDL clearance in the experimental model.^77^ DEHP also increases triglyceride and very-low-density lipoprotein (VLDL) levels in female albino mice, further suggesting lipid metabolism disruption.^78^ In peritoneal dialysis (PD) patients with serum DEHP levels ≥0.0687 mg/mL, statin treatment failed to reduce CVD risk, providing preliminary evidence that warrants prospective evaluation.^79^ *In vitro*, DEHP inhibited simvastatin-induced transient receptor potential vanilloid type 1 (TRPV1)/Ca^2+^ signaling in endothelial cells (ECs), reduced nitric oxide (NO) bioavailability, and impaired EC migration and proliferation. DEHP also abrogated the anti-inflammatory effects of simvastatin on TNF-α-induced adhesion molecule expression (e.g., intercellular adhesion molecule-1 [ICAM-1] and vascular cell adhesion molecule-1 [VCAM-1]), further compromising vascular homeostasis.^79^ Collectively, these findings generate a hypothesis regarding possible DEHP-related interference with statin responses; they do not provide a basis for modifying statin therapy in patients with CKD.

In summary, the mechanisms linking phthalates to CKD span intracellular stress cascades (oxidative stress, regulated cell death, and fibrogenic remodeling), systemic metabolic and gut axis perturbations, developmental programming, and extra-renal amplification through environmental synergy and therapeutic interference (Figure 2). Figure 2 should be interpreted as a proposed evidence framework rather than a deterministic causal cascade. Color-coded evidence labels distinguish the predominant evidence context: blue denotes human observational evidence, green denotes mammalian experimental evidence, purple denotes non-mammalian or *in vitro* evidence, and gray denotes indirect inference or extra-renal evidence. These labels indicate the primary evidence context of each component and should not be interpreted as a quantitative ranking of evidence strength. The relative contribution of each pathway to human CKD onset and progression remains to be determined through appropriately designed longitudinal and interventional research.

## Clinical and research implications

The accumulated mechanistic evidence for phthalate-associated CKD—spanning oxidative stress, MEHP-related nephrotoxicity, regulated cell death, and downstream transport/EMT-fibrogenic remodeling—together with population-level observations provides a rationale for further translational research on population-level exposure reduction, biomarker validation, and potential treatment-pathway interactions (Figure 3). Notably, all clinical implications described below remain preliminary and preclinical. None of the current biomarkers or intervention strategies has been clinically validated for routine CKD management, and all translational statements in this review are framed as research hypotheses rather than confirmed clinical recommendations.

**Figure 3:**
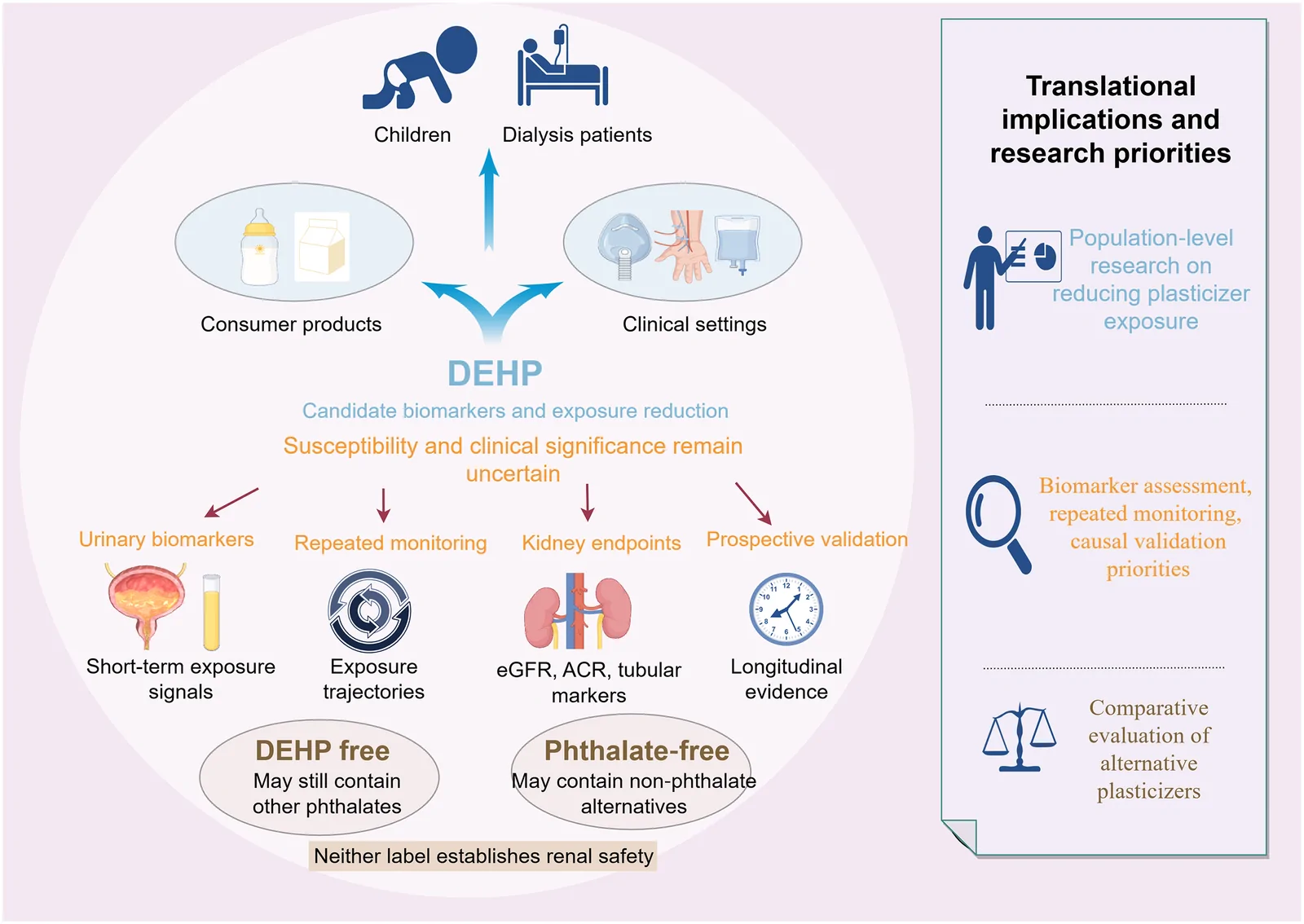
Translational implications and research priorities for phthalate-associated kidney outcomes DEHP exposure may occur through consumer products and clinical settings, with children and dialysis patients representing populations in which exposure and renal susceptibility warrant further investigation. Urinary phthalate metabolites may serve as candidate biomarkers for population-level exposure assessment, but their ability to reflect sustained exposure and predict individual CKD outcomes remains unvalidated. Repeated biomonitoring may help characterize exposure trajectories, while kidney endpoints, including estimated glomerular filtration rate (eGFR), albumin-to-creatinine ratio (ACR), and tubular-injury markers, should be evaluated in longitudinal studies. Prospective validation is needed to clarify temporality, causal relationships, and whether exposure reduction is accompanied by improvement or stabilization of renal outcomes. The figure also emphasizes the need for population-level research on reducing plasticizer exposure and for comparative evaluation of alternative plasticizers. DEHP-free products may still contain other phthalate esters, whereas phthalate-free products may contain chemically distinct non-phthalate alternatives; neither label establishes renal safety. These translational implications represent research priorities rather than validated tools for individual CKD risk stratification or treatment modification.

### Exposure reduction in high DEHP-exposure populations

In children, developmental immaturity increases renal susceptibility to phthalate toxicity, while medical devices and daily consumer products create frequent high-exposure scenarios. Neonates in neonatal intensive care units (NICUs) exhibit elevated phthalate exposure from respiratory and intravenous tubing, exacerbated by immature metabolic and excretory function.^16^ Pediatric transplant cohorts show higher urinary phthalate metabolites than healthy children, and early-life high phthalate exposure is epidemiologically correlated with long-term albuminuria elevation.^40^^,^^80^ These observations generate the research hypothesis that targeted exposure reduction and longitudinal renal follow-up represent research priorities for evaluating long-term renal risk in vulnerable pediatric population. In advanced CKD patients, impaired renal clearance and repeated dialysis-related plastic device contact facilitate DEHP bioaccumulation.^38^ Existing pharmacokinetic data demonstrate that non-PVC or low-leaching tris(2-ethylhexyl) trimellitate (TOTM)-modified dialysis tubing significantly reduces intra-dialytic DEHP release and patient DEHP retention compared with conventional DEHP-plasticized PVC tubing.^81^ These findings suggest a potential protective trend; however, clinical outcome evidence is still lacking. Reducing medical phthalate leakage may serve as a future preventive research direction for dialysis populations. Barriers including cost, supply limitations, and material accessibility require further health-system investigation.

### Biomarker assessment, repeated monitoring, and causal validation priorities

Translational progress in phthalate-associated CKD depends on methodological advances in exposure assessment and study design. Secondary oxidized DEHP metabolites generally offer analytical advantages, including relatively high stability and low contamination interference, and may serve as useful indicators of recent exposure. Their ability to reflect cumulative exposure requires repeated measurements and prospective validation.^82^^,^^83^ Toxicogenomic profiles further suggest that oxidative and xenobiotic metabolism signatures may be associated with phthalate-related renal stress.^84^ Nevertheless, urinary phthalate metabolites have not been formally validated as clinical risk-stratification biomarkers for CKD. CKD-related physiological disturbances may interfere with conventional correction methods, and creatinine or specific-gravity normalization may produce directionally biased associations in CKD populations compared with the general population.^85^^,^^86^ Covariate-adjusted models incorporating renal function, age, and sex may improve exposure assessment in research settings, but such approaches cannot yet be applied to individual clinical risk judgment.^80^

Single spot urine measurements poorly capture intra-individual exposure variability and may be difficult to interpret in CKD because declining renal function can alter metabolite excretion. Future studies should move beyond cross-sectional spot samples by incorporating serial urine collections, 24-h urine samples, or parallel plasma metabolite profiling when feasible. These designs may help distinguish sustained external exposure from changes in renal clearance and provide exposure trajectories that cannot be captured by single measurements.

Observational associations cannot establish whether phthalate exposure precedes renal injury or whether diminished renal excretion in CKD alters measured urinary metabolite concentrations. Directed acyclic graphs and longitudinal cohort designs with repeated assessment of renal function may help strengthen causal inference. Instrumental variable approaches, including Mendelian randomization analyses when robust and biologically relevant genetic instruments are available, may also be considered. Natural experiments, such as dialysis centers transitioning to low-leaching tubing or pediatric units reducing the use of PVC-containing medical devices, may provide opportunities to evaluate whether exposure reduction is accompanied by improvement or stabilization of validated renal endpoints. Randomized intervention studies are currently premature. Priority should instead be given to well-characterized prospective cohorts with standardized exposure assessment, appropriate follow up, and predefined renal and mechanistic outcomes.

### Implications for phthalate alternatives

Global regulatory and market shifts away from DEHP toward alternative plasticizers do not necessarily eliminate potential renal risk, as emerging epidemiological data suggest that certain substitutes may retain biological activity relevant to nephrotoxicity, while mixed organic pollutant exposure may jointly influence renal disease outcome.^87^^,^^88^ These categories should not be conflated. A DEHP-free product does not contain DEHP specifically but may still contain other phthalate esters. A phthalate-free product excludes phthalate esters but may contain chemically distinct, non-phthalate alternative plasticizers, such as DEHTP, TOTM, or citrate esters. The exposure sources, metabolism, toxicological profiles, and renal evidence bases of these alternatives may differ from those of DEHP; therefore, neither “DEHP-free” nor “phthalate-free” labeling establishes renal safety. This evidence highlights the research perspective that phthalate-free products are not necessarily equivalent to “renal-safe” products. Current findings support population health research on reducing comprehensive plasticizer exposure and evaluating alternative plasticizers, rather than guiding individualized clinical intervention.

## Conclusion and future perspective

Overall, existing evidence supports a biologically plausible but incompletely validated mechanistic framework. Endogenous metabolite accumulation induces local renal stress and inflammatory responses, further triggering abnormal cell damage and dysfunction. These pathological alterations subsequently promote EMT and excessive ECM accumulation, which may contribute to renal tubular dysfunction and fibrotic remodeling in experimental settings. This mechanistic chain is also directionally consistent with population-level exposure associations, particularly among high-sensitivity/high-contact groups such as dialysis patients and children. From a clinical translation perspective, this review organizes mechanistic modules into testable translational research questions, including source control for exposure related to medical devices, validation of metabolites and early kidney-injury indicators as candidate research biomarkers, and the exploration of adjunctive interventions in populations with high oxidative/inflammatory burdens, while emphasizing that phthalate-free does not necessarily mean “risk-free” and that substitutes still require ongoing evaluation. Nevertheless, several key limitations remain. First, causal inference is still insufficient: most studies are observational, and exposure assessment is influenced by individual metabolic differences as well as urine dilution and standardization methods, which may over- or under-estimate the true strength of associations. Second, the relative contributions across renal segments, time windows, and metabolite profiles (e.g., DEHP metabolites versus those derived from alternative plasticizers) have not been fully disentangled, and the temporal relationships among mechanisms remain uncertain. Third, there is no unified, clinically feasible framework to address multi-pollutant co-exposure and mixture effects.

Future work should focus on: conducting longitudinal follow-up and prospective intervention studies (especially in dialysis centers and pediatric high-risk cohorts), integrating longitudinal metabolite trajectories, mechanism-oriented biomarker panels, and standardized sampling strategies; and further establishing reproducible dose-response models and alternative safety assessment systems under mixed-exposure conditions. These steps will help advance the field from “association and biological plausibility” toward causal evidence capable of guiding clinical decisions, and toward more generalizable risk-management approaches.

## Acknowledgments

All figures in this article were drawn by Figdraw. (https://www.figdraw.com/static/index.html#/).

## Author contributions

C.W., writing – original draft and writing – review and editing; H.C., writing – original draft and writing – review and editing; Q.L., writing – original draft; Y.T., writing – original draft; Z.L., writing – original draft; S.C., writing – original draft; S.W., writing – original draft.

## Declaration of interests

The authors declare that they have no known competing financial interests or personal relationships that could have appeared to influence the work reported in this paper.
